# Long Withdrawal of Methylphenidate Induces a Differential Response of the Dopaminergic System and Increases Sensitivity to Cocaine in the Prefrontal Cortex of Spontaneously Hypertensive Rats

**DOI:** 10.1371/journal.pone.0141249

**Published:** 2015-10-28

**Authors:** Maurício dos Santos Pereira, Matheus Figueiredo Sathler, Thais da Rosa Valli, Richard Souza Marques, Ana Lucia Marques Ventura, Ney Ronner Peccinalli, Mabel Carneiro Fraga, Alex C. Manhães, Regina Kubrusly

**Affiliations:** 1 Laboratório de Neurofarmacologia, Departamento de Fisiologia e Farmacologia, Instituto Biomédico, Universidade Federal Fluminense, Niterói, RJ, Brazil; 2 Laboratório de Neuroquímica, Departamento de Neurobiologia, Instituto de Biologia, Universidade Federal Fluminense, Niterói, RJ, Brazil; 3 Laboratório de Neurofisiologia, Departamento de Ciências Fisiológicas, Instituto de Biologia Roberto Alcantara Gomes, Centro Biomédico, Universidade do Estado do Rio de Janeiro, Rio de Janeiro, RJ, Brazil; University of Colorado, UNITED STATES

## Abstract

Methylphenidate (MPD) is one of the most prescribed drugs for alleviating the symptoms of Attention Deficit/Hyperactivity Disorder (ADHD). However, changes in the molecular mechanisms related to MPD withdrawal and susceptibility to consumption of other psychostimulants in normal individuals or individuals with ADHD phenotype are not completely understood. The aims of the present study were: (i) to characterize the molecular differences in the prefrontal dopaminergic system of SHR and Wistar strains, (ii) to establish the neurochemical consequences of short- (24 hours) and long-term (10 days) MPD withdrawal after a subchronic treatment (30 days) with Ritalin® (Methylphenidate Hydrochloride; 2.5 mg/kg orally), (iii) to investigate the dopaminergic synaptic functionality after a cocaine challenge in adult MPD-withdrawn SHR and Wistar rats. Our results indicate that SHR rats present reduced [^3^H]-Dopamine uptake and cAMP accumulation in the prefrontal cortex (PFC) and are not responsive to dopaminergic stimuli in when compared to Wistar rats. After a 24-hour withdrawal of MPD, SHR did not present any alterations in [^3^H]-Dopamine Uptake, [^3^H]-SCH 23390 binding and cAMP production; nonetheless, after a 10-day MPD withdrawal, the results showed a significant increase of [^3^H]-Dopamine uptake, of the quantity of [^3^H]-SCH 23390 binding sites and of cAMP levels in these animals. Finally, SHR that underwent a 10-day MPD withdrawal and were challenged with cocaine (10 mg/kg i.p.) presented reduced [^3^H]-Dopamine uptake and increased cAMP production. Wistar rats were affected by the 10-day withdrawal of MPD in [^3^H]-dopamine uptake but not in cAMP accumulation; in addition, cocaine was unable to induce significant modifications in [^3^H]-dopamine uptake and in cAMP levels after the 10-day withdrawal of MPD. These results indicate a mechanism that could explain the high comorbidity between ADHD adolescent patients under methylphenidate treatment and substance abuse in adult life.

## Introduction

Attention deficit/hyperactivity disorder (ADHD) is one of the most common childhood diseases, affecting approximately 3–7% of worldwide school-aged children. Although there is a tendency for symptoms to disappear over time, some individuals continue to present them at adulthood [1, 2]. Genetic factors support the hypothesis of dopamine hypofunction in ADHD as a primary cause of behavioral symptoms such as hyperactivity, impulsiveness and deficient sustained attention [3, 4].

The prefrontal cortex (PFC) is involved with cognitive processes thought to comprise executive function, which includes impulse control, response inhibition, attention, working memory, cognitive flexibility, planning, judgment, and decision-making [5]. Some of these functions depend on dopaminergic neuronal activity and are described as inefficient in ADHD [6, 7]. It has been suggested that behavioral disturbances associated with ADHD are the result of an imbalance between noradrenergic and dopaminergic systems in the PFC [8].

Several reports describe Spontaneously Hypertensive Rats (SHR) as one of the most appropriate animal models to study ADHD since SHR are hyperactive and show defective sustained attention in behavioral tasks [9, 10]. SHR exhibit modifications on the dopaminergic system similar to those observed in individuals with ADHD, such as a lower expression of the D4 receptor gene (DRD4) in the prefrontal cortex in addition to reduced dopamine release [11, 12].

Dopamine transporter (DAT) activity is also believed to be critically involved in the dopaminergic dysfunction associated with ADHD and, therefore, should constitute a crucial molecular target for the treatment of this disease [13]. Several lines of research indicate that SHR have DAT expression levels above its control strain in the PFC and striatum [10]. However, the dopaminergic transmission is significantly reduced [14]. Similarly, patients with ADHD present a larger DAT expression than normal individuals, although its functionality seems to be decreased [15].

Methylphenidate (MPD) is one of the most prescribed drugs for alleviating the symptoms of ADHD. The efficacy of this treatment has been widely described before [16]. Evidence strongly indicates that the effects of MPD in improving behavioral deficits in ADHD children are the result of dopaminergic and noradrenergic modulation in the PFC [17]. These findings could be related to MPD effects on the PFC, regulating D1-like dopaminergic and α2-noradrenergic receptors [18]. Accordingly, it has been observed that acute and chronic exposure to MPD in the nucleus accumbens are able to modify neuronal firing rates through D1- and D2-like receptor activation [19], and that MPD-withdrawal is able to increase the neural activity in the dorsal midbrain [20].

Despite the benefits of MPD treatment in ADHD, several reports express concern about the misuse of MPD associated with drug abuse [4, 21]. It is largely accepted that not only MPD acts on dopamine transporter and inhibits dopamine reuptake, but also that it promotes rewarding effects by a mechanism similar to cocaine [22, 23]. Moreover, it has been suggested that long exposure to MPD in juvenile animal models for ADHD (such as SHR) enhances cocaine self-administration and vulnerability to drug abuse [13, 24, 25].

However, the molecular mechanisms of the dopaminergic system affected by MPD that could be involved in the susceptibility to other psychostimulants are not completely understood. So, in the present study we used SHR and Wistar rats to characterize dopamine uptake and cAMP production in both strains. We also evaluated dopaminergic changes in the PFC induced by chronic Ritalin® treatment (Methylphenidate Hydrocloride), followed by a short-term (24 hours) and a long-term (10 days) MPD withdrawal. Finally, we challenged SHR and Wistar rats with a single dose of cocaine in order to analyze the neurochemical effects of this psychostimulant on a PFC dopaminergic system previously exposed to MPD.

## Material and Methods

### Ethics Statement

All experiments were carried out under institutional approval of the Animal Care and Use Committee of the Universidade do Estado do Rio de Janeiro (CEUA/001/2013), in accordance with Brazilian Law n° 11.794/2008 and with the Guide for the Care and Use of Laboratory Animals as adopted and promulgated by the National Institutes of Health. All efforts were done to minimize the number of animals used and their suffering.

### Animals

Spontaneously Hypertensive Rats (SHR) and Wistar rats were maintained in our institutional animal care facility in Universidade Federal Fluminense (Niterói, RJ, Brazil). Wistar rats represent an outbred heterogeneous control model for SHR for neurochemical analyzes. Original breeding stock was obtained from Universidade do Estado do Rio de Janeiro (Rio de Janeiro, RJ, Brazil). SHR were mated within the same brood to ensure strain isogeny. Therefore male SHR from brood A1 would only mate with female from brood A1. However, to ensure Wistar heterogeneity, male rats only mated with female rats from different broods. Thereby, a male Wistar from brood B1 could never mate with a female from brood B1 and their descendants (B2 onwards). The day of birth was considered postnatal day 0 (PN0).

At weaning (PN21; 30 g), male rats from each brood were separated from their progenitors and housed 2–4 per cage (49 × 34 × 16 cm polypropylene cage containing wood shavings) in a temperature-controlled (22±1**°**C) room on a 12-h light/dark cycle (lights on at 8:00 a.m.), with unlimited access to food and water.

### Materials

Methylphenidate hydrochloride (MPD; Ritalin®) was produced by Novartis AG (Basel, Switzerland). Cocaine hydrochloride, R(+)-SCH 23390 hydrochloride (SCH 23390) and Dopamine Hydrochloride were purchased from Sigma Aldrich Co. (St. Louis. MO, USA). [^3^H]-Dopamine (specific activity: 56.8 Ci/mmol) and [^3^H]-cAMP (specific activity: 28.1 Ci/mmol) were acquired from PerkinElmer (Massachusetts, USA). [^3^H]-SCH23390 (specific activity: 70.3 Ci/mmol) was acquired from Amersham (USA). All other chemicals were of the highest purity obtainable from regular commercial sources. Each experiment was performed at least 3 times.

### Methylphenidate and Cocaine Treatment Protocol

**Fig 1:**
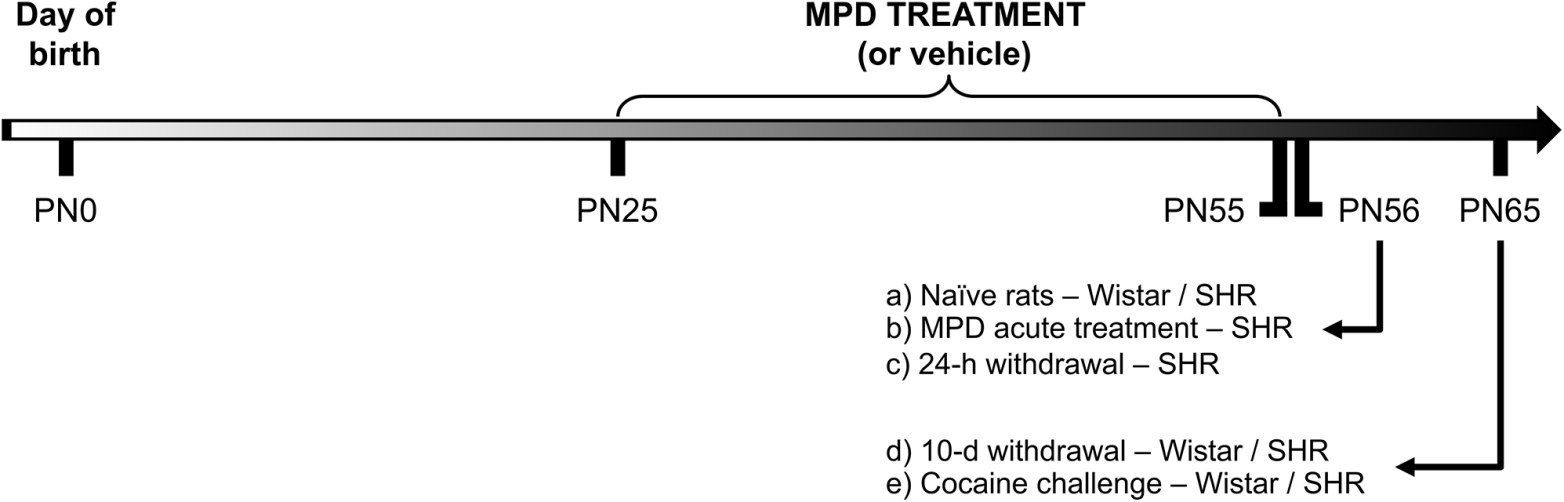
Experimental timeline.

A sample of PN25 (30–40 g) SHR and Wistar rats were treated daily (around 4:00 p.m. in a sound-attenuated room next to the animal facility) with MPD (2.5 mg/kg orally; diluted in drinking water and given by gavage: 2 ml/kg) for 30 consecutive days. This specific dose is capable of alleviating motor dysfunction in rats exposed to manganese during development, an animal model of induced ADHD phenotype [26]. Control rats received only drinking water (vehicle). After 30 days of treatment with MPD or vehicle (PN55; 250 g), animals were divided into the following experimental groups (Fig 1): a) Naïve rats; b) Acute treatment with MPD after chronic treatment with vehicle; c) 24-hour withdrawal from chronic treatment with MPD (PN56); d) 10-day withdrawal from chronic treatment with MPD (PN65); e) 10-day withdrawal from chronic treatment with MPD + single dose of cocaine (10 mg/kg, i.p.) at PN65, 20 min prior to euthanasia (control group received corresponding volume of saline 0.9% i.p.). At the end of treatment or withdrawal, rats were anesthetized by isofluorane inhalation and decapitated by guillotine. Brains were rapidly dissected and kept on cold (4°C) physiological saline solution to perform neurochemical experiments. Specific samples sizes used in each experiment will be provided in the Fig legends.

### [^3^H]-Dopamine Uptake

Samples of prefrontal cortex from SHR and Wistar rats were incubated for 60 min in 1 mL of MEM (minimum essential medium) containing 1 μCi [^3^H]-Dopamine (1.2 x 10^-6^M) buffered to pH 7.4 with 20 mM HEPES at 37°C. The medium was removed and the tissue washed four times with 3 mL of cold Hanks 4 solution. This procedure was sufficient to wash out the free radioactivity (not taken up by the tissue). After the washes, 1 mL of Milli-Q water was added to the tissue to disrupt the cells. Following successive freeze-thaw cycles, cellular radioactivity was assayed using a scintillation counter. In a separate experiment, a time-course of [^3^H]-Dopamine uptake was performed at the following time points (in min): 10, 30, 60, 90 and 120.

### Cyclic AMP Assay

The prefrontal cortex samples were pooled, cut into 2 mm^2^ pieces and incubated for 60 min at 37°C in minimal essential medium (MEM) buffered with 20 mM HEPES (pH 7.4) containing 100 μM ascorbic acid, 100 μM pargyline and 0.5 mM IBMX (a phosphodiesterase inhibitor). In other experiments, exogenous dopamine (Dopamine Hydrochloride, 200 μM) or SCH 23390 (50 μM) was added prior to incubation. The reaction was stopped by adding 10% trichloroacetic acid (final concentration). The cAMP was purified and assayed by previously described methods [27].

### Western Immunoblotting

Samples of the prefrontal cortex were homogenized with RIPA buffer containing a protease inhibitor cocktail. Protein concentration was estimated by the BCA Protein Assay Kit (Pierce) method. Samples were diluted in buffer composed of 10% glycerol (v/v), 1% ß-mercaptoethanol, 3% SDS, and 62.5 mM Tris base, and boiled for 5 min. Approximately 30 μg of protein from each sample was electrophoresed in 10% SDS–PAGE and transferred to nitrocellulose membranes (ECL-Hybond). The membranes were washed with Tween 20 Tris-buffered saline (TBS-T) and blocked for 1.5 h with 5% skim milk in TBS-T. The membranes were incubated with the anti-DAT (1:500 in TBS-T; Sigma) or anti-D1R (1:500 in TBS-T; Sigma) overnight at 4°C, rinsed in TBS-T and incubated with anti-rabbit (for DAT) and anti-mouse (for D1R) peroxidase conjugated secondary antibody for 1.5 h at room temperature. Following three washes in TBS-T (10 min each), the labeling was detected with an ECL kit (Amersham). Blots were reprobed with anti-tubulin antibody (1:25,000 in TBS-T, Sigma) for 1 h at room temperature, rinsed in TBS-T and incubated with anti-mouse peroxidase conjugated secondary antibody for 45 min at room temperature. Following three TBS-T washes (10 min each), the labeling was detected with the ECL kit. Band intensities were analyzed by using Quantity One 4–6 software (Bio-Rad Laboratories Inc). The Arbitrary Units (A.U.) used as densitometry data represent pixel analysis from band intensities of DAT or D1R divided by their respective tubulin bands intensities.

### [^3^H]-SCH 23390 Binding Assay

The [^3^H]-SCH 23390 binding assay protocol was carried out as previously described [28]. Briefly, SHR prefrontal cortex samples were washed three times with CMF and the cells lysed in a 5 mM Tris–HCl, 5 mM MgCl_2_ buffer (pH 7.4) at 4°C. The cells were disrupted using a Dounce homogenizer followed by centrifugation at 15,000×g, for 30 min. The resulting membrane pellet was resuspended in binding buffer (50 mM Tris–HCl, pH 7.4, containing 120 mM NaCl, 5 mM KCl, 1.5 mM CaCl_2_, 4 mM MgCl_2_ and 1 mM EDTA). Samples containing approximately 0.5 mg of protein were incubated with [^3^H]-SCH 23390 (70.3 Ci/mmol) in a final volume of 0.2 mL. (+)-SCH 23390 was added at the final concentration of 5 mM to determine nonspecific binding. Assay tubes were incubated at room temperature for 1 h and the reaction was terminated by rapid filtration through GF/C glass fiber filters pretreated with 0.3% polyethyleneimine. Radioactivity bound to the filters was quantified by liquid scintillation spectroscopy. Binding assays were performed in duplicate and in four different experiments.

### Statistical Analysis

Data are compiled as means ± S.E.M. The Kolmogorov–Smirnov one sample test (K–S) was used to assess the normality of the distributions of each of the variables. In order to compare [^3^H]-Dopamine uptake between naïve SHR and Wistar rats, unpaired Student t tests were used. The analysis of [^3^H]-Dopamine uptake at different time points in both strains was carried out by using a two-way ANOVA: Strain (SHR or Wistar) and Time Point (10, 30, 60, 90 and 120 min; different tissue samples at each time point) were used as between-subjects factors. Differences in cAMP accumulation between strains in a basal condition and after exogenous dopamine stimulation were also analyzed by a two-way ANOVA: Strain (SHR or Wistar) and Condition (basal or dopamine-stimulated; different samples in each condition) were used as between-subjects factors. A one-way ANOVA was used to analyze the effect of MPD administration and withdrawal in SHR animals: Group (Control, acute MPD, 24 h withdrawal from MPD and 10 d withdrawal from MPD; different animals in each group) was considered the between-subjects factor. Two-way ANOVAs having Treatment (Control or MPD) and Duration of Withdrawal (24 h or 10 d; different animals at each time point) as between-subjects factors were used for the following analyses: DAT expression, D1R expression and [^3^H]-SCH 23390 binding. Differences in cAMP accumulation among SHR groups as a function of MPD treatment, SCH use, and duration of withdrawal were also analyzed using a two-way ANOVA: Group (Control, SCH, MPD and MPD + SCH) and Duration of Withdrawal (24 h or 10 d) were used as between-subjects factors. Finally differences between SHR and Wistar rats regarding [^3^H]-Dopamine uptake and cAMP accumulation as a function of MPD treatment and COC use were analyzed by two-way ANOVAs: Strain (Wistar and SHR) and Group (Control, COC, MPD and MPD + COC) were used as between-subjects factors. Unpaired Student t tests and Fisher’s Protected Least Significant Difference (FPLSD) tests were used post hoc. For main effects and interactions, significance is assumed at the level of p < 0.05.

## Results

### Comparison of [^3^H]-Dopamine Uptake and cAMP accumulation in SHR and Wistar rats

**Fig 2:**
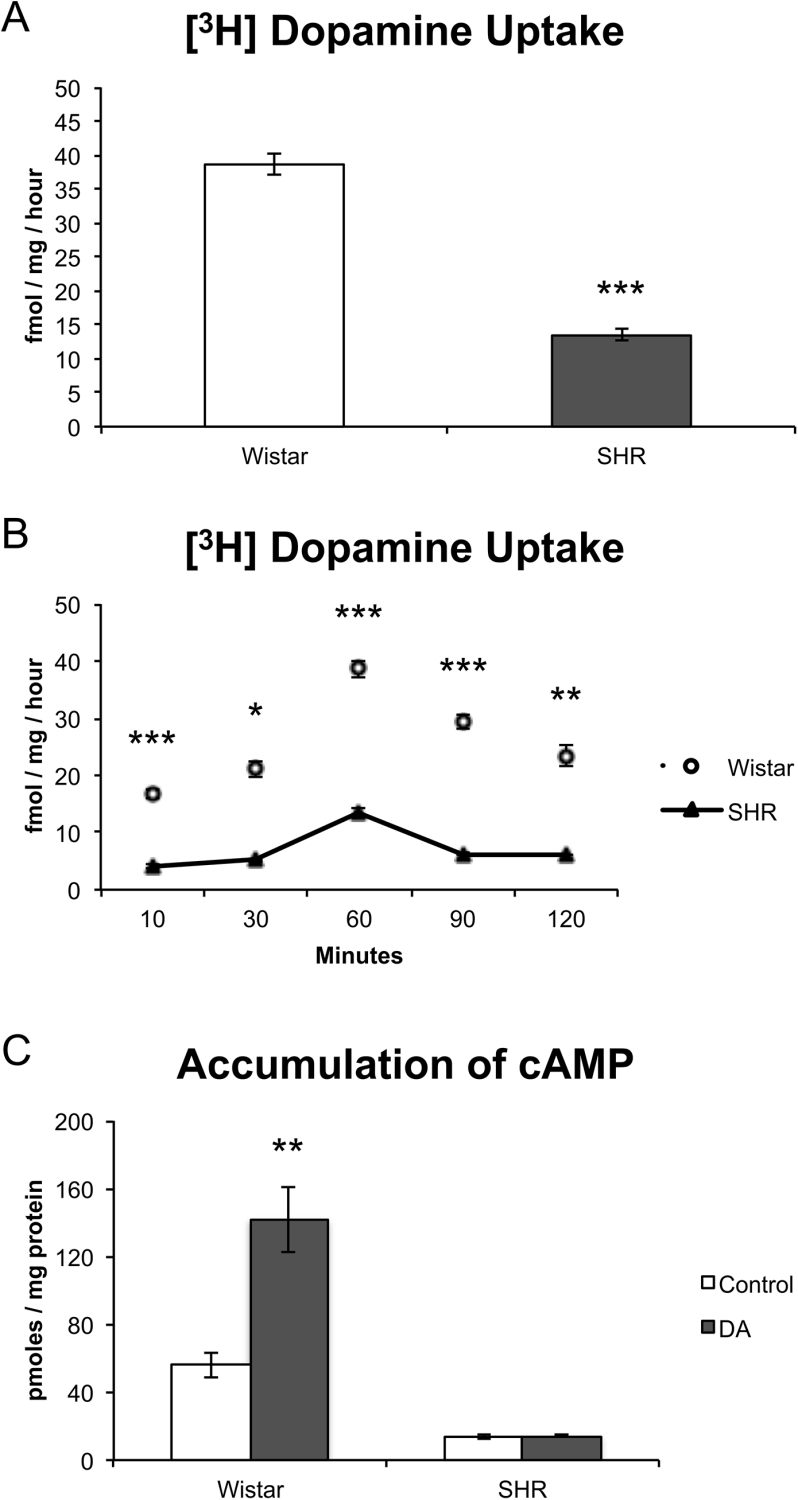
Characterization of [^3^H]-Dopamine uptake and cAMP accumulation in naïve Wistar and SHR rats. (A) [^3^H]-Dopamine uptake after 60 min in the PFC (n = 6–8). (B) Time curve of [^3^H]-Dopamine uptake (n = 3–6). (C) cAMP levels in PFC slices in basal conditions and after exogenous dopamine stimulation (200 μM) (n = 4–8). Results are expressed as means ± S.E.M. A and B: * = p < 0.05; ** = p < 0.01; *** = p < 0.001, SHR vs. Wistar. C: ** = p < 0.01, Control vs. DA.

[^3^H]-Dopamine uptake in slices of PFC showed a significant reduction of basal dopamine uptake of SHR in comparison to Wistar rats (t = 14.5, df = 10, p < 0.001; Fig 2A). The analysis of [^3^H]-Dopamine uptake at different time points indicated that SHR rats present a diminished dopamine uptake in comparison to Wistar ones (Strain effect: F = 543, d.f. = 1, p < 0.001; Fig 2B).

The pattern held when cAMP accumulation was analyzed, with SHR animals exhibiting decreased basal cAMP levels when compared to Wistar (Strain effect: F = 88, d.f. = 1, p < 0.001 Fig 2C). Surprisingly, the effect of stimulation with exogenous dopamine was specific to Wistar rats (Strain × Condition interaction: F = 22, d.f. = 1, p < 0.001), which presented a significant increase in cAMP levels (t = 4.9, p = 0.001), markedly differing from SHR animals, which did not present any variation in cAMP accumulation (p > 0.05).

### [^3^H]-Dopamine Uptake after Chronic or Acute Treatment with MPD

**Fig 3:**
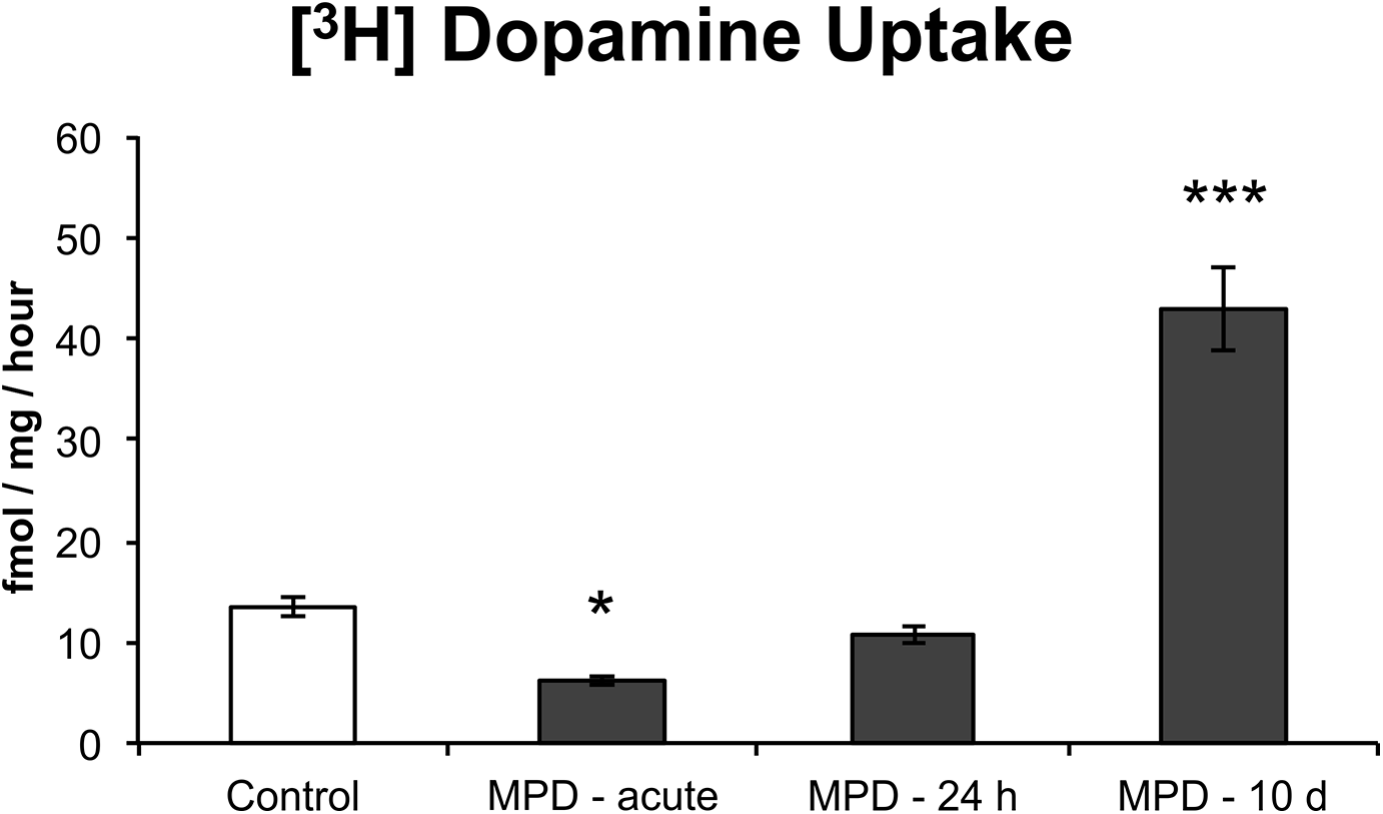
Analysis of SHR [^3^H]-Dopamine uptake. [^3^H]-Dopamine uptake was analyzed 24 h (PN56) and 10 days (PN65) after chronic treatment with MPD, as well as 30 min after a single treatment with MPD in vehicle-treated animals (n = 5–6). Results are expressed as means ± S.E.M. * = p < 0.05; *** = p < 0.001, vs. Control.

As previously described, MPD (Ritalin®) is a potent blocker of catecholamine reuptake [23]. We have examined [^3^H]-Dopamine uptake in the PFC of SHR animals 30 min after a single MPD administration, as well as 24 h and 10 days following the last MPD administration of the treatment protocol (Fig 3). A significant Group effect was observed (F = 63, d.f. = 3, p < 0.001): At 30 min post-acute administration, a significant reduction in uptake was observed (FPLSD: p = 0.021). No difference between the control group and the MPD group was observed at 24 hours of withdrawal. However, [^3^H]-Dopamine uptake was significantly increased after a 10-day withdrawal of MPD when compared to the controls (FPLSD: p < 0.001).

### Effects of MPD on DAT and D1R Expression

**Fig 4:**
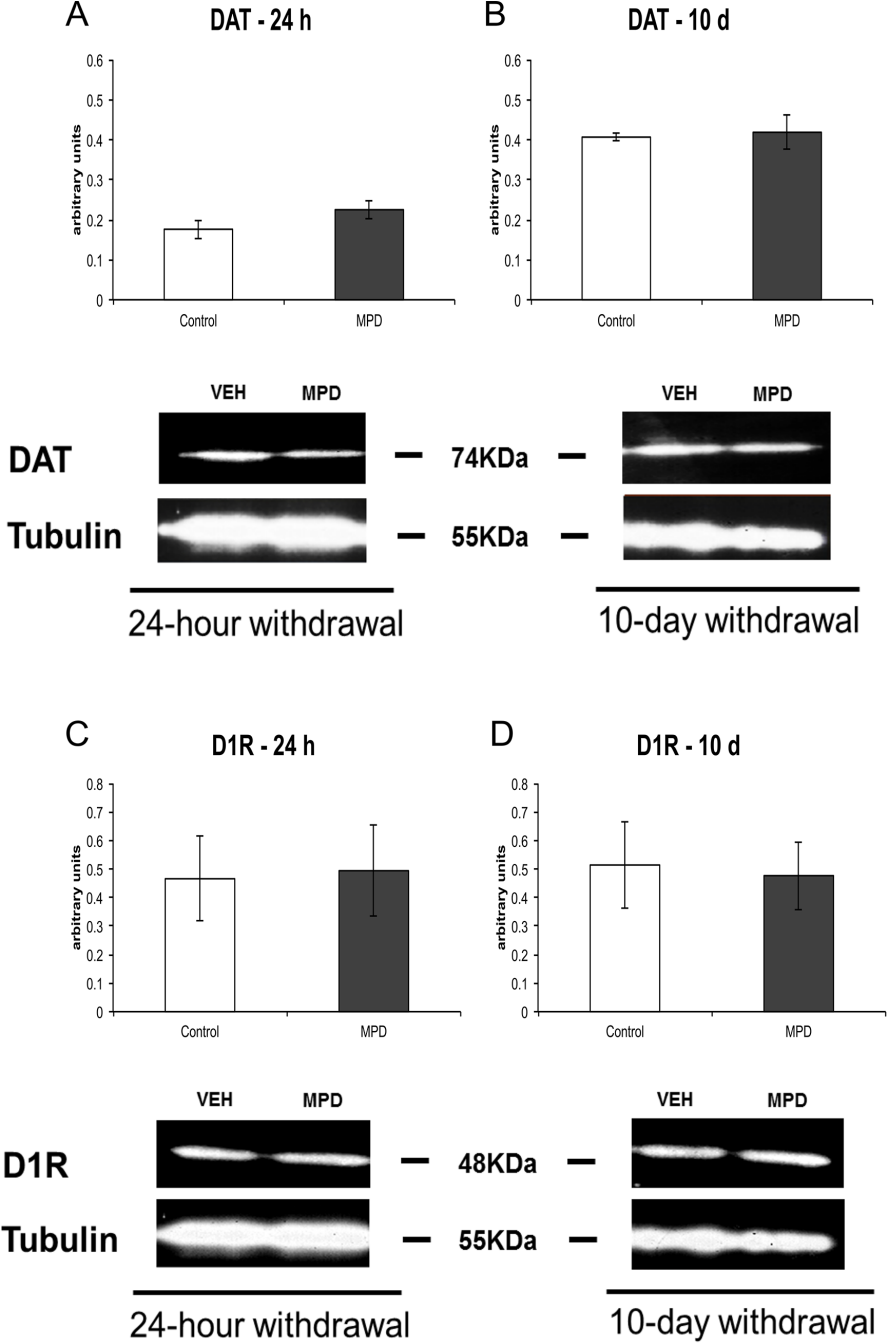
Western Immunoblotting for DAT and D1R. PFC samples of SHR rats were incubated with anti-DAT (1:500) or D1R (1:500) and anti-Tubulin (1:25000) at PN56 (A and C) or PN65 (B and D) after chronic MPD treatment. Results of densitometry indicated in arbitrary units (n = 3). Results are expressed as means ± S.E.M.

To determine whether MPD effects on [3H]-Dopamine uptake required changes in DAT or D1R expression in SHR animals, we performed a Western Immunoblotting assay (Fig 4). No differences were observed in DAT or D1R as a result of MPD treatment (p > 0.05).

### [^3^H]-SCH 23390 Binding and cAMP Accumulation after MPD withdrawal

**Fig 5:**
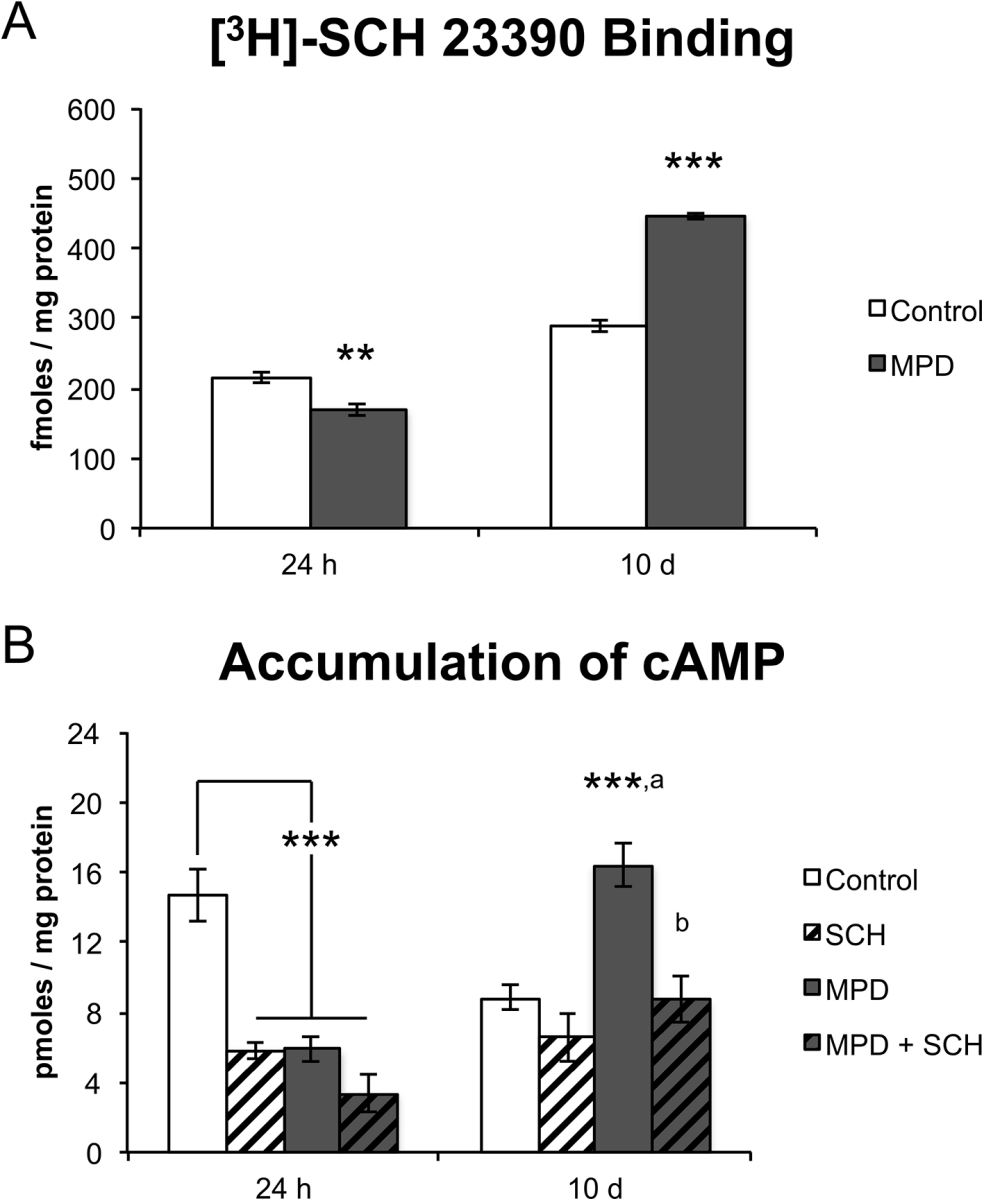
[^3^H]-SCH 23390 binding and cAMP accumulation after MPD withdrawal in SHR PFC. (A) [^3^H]-SCH 23390 binding in the 24-h (PN56) and 10-day (PN65) MPD withdrawal and control groups (n = 4). (B) Basal cAMP accumulation in the 24-h (PN56) and 10-day (PN65) withdrawal group and after SCH 23390 exposure (50 μM; n = 4–7). Results are expressed as means ± S.E.M. ** = p < 0.01; *** = p < 0.001, vs. Control. a = p < 0.001, vs. SCH. b = p < 0.001, vs. MPD.

We carried out D1-like binding assays in isolated membrane fractions of SHR cortical cells using [^3^H]-SCH 23390 as the specific D1-like antagonist to verify whether specific D1-like receptor expression on the cell surface would be modified after MPD treatment (Fig 5A). We observed a significant Treatment × Time Point interaction (F = 179, d.f. = 1, p < 0.001) that is explained by the fact that, after a 24-h withdrawal from MPD, a significant (t = 4.2, p = 0.006) reduction in binding was observed when compared to controls, while, after a 10-day period of withdrawal, the opposite was true (t = 15.1, p < 0.001). The final concentration of [^3^H]-SCH23390 was 1.25 nM to establish the maximum of D1R binding sites, as previously described by Kirouac and Ganguly [29].

Considering that the D1-like receptor binding is affected, we next verified whether dopaminergic receptor function could be modulated after MPD withdrawal (Fig 5B). A significant Group × Duration of Withdrawal interaction (F = 20, d.f. = 3, p < 0.001) was observed regarding cAMP accumulation. The 24 h withdrawal data indicated that the SCH, MPD and MPD + SCH groups presented significant (FPLSD: p < 0.001 in all pairwise comparisons) reductions when compared to controls, although no differences were observed among them. A markedly different pattern was observed at 10 days of withdrawal: cAMP levels were significantly (FPLSD: p < 0.001 in all pairwise comparisons) increased in the MPD group when compared to all other groups, while no differences existed among these groups (Control, SCH and MPD + SCH).

### Effects of 10 Days of MPD withdrawal and Cocaine Challenge in SHR and Wistar Rats

SHR and Wistar rats treated with MPD for 30 days that underwent a 10-day withdrawal were submitted to a single administration of cocaine (10 mg/kg i.p.) 20 minutes prior to euthanasia (PN65) in order to investigate the neurochemical effects of cocaine in a system previously exposed to MPD: [^3^H]-Dopamine uptake and cAMP accumulation were analyzed for this assessment. A significant Strain × Group interaction was observed regarding both [^3^H]-Dopamine uptake (F = 4.7, d.f. = 3, p = 0.005) and cAMP accumulation (F = 8.5, d.f. = 3, p < 0.001). We therefore decided to analyze the data of each strain separately.

**Fig 6:**
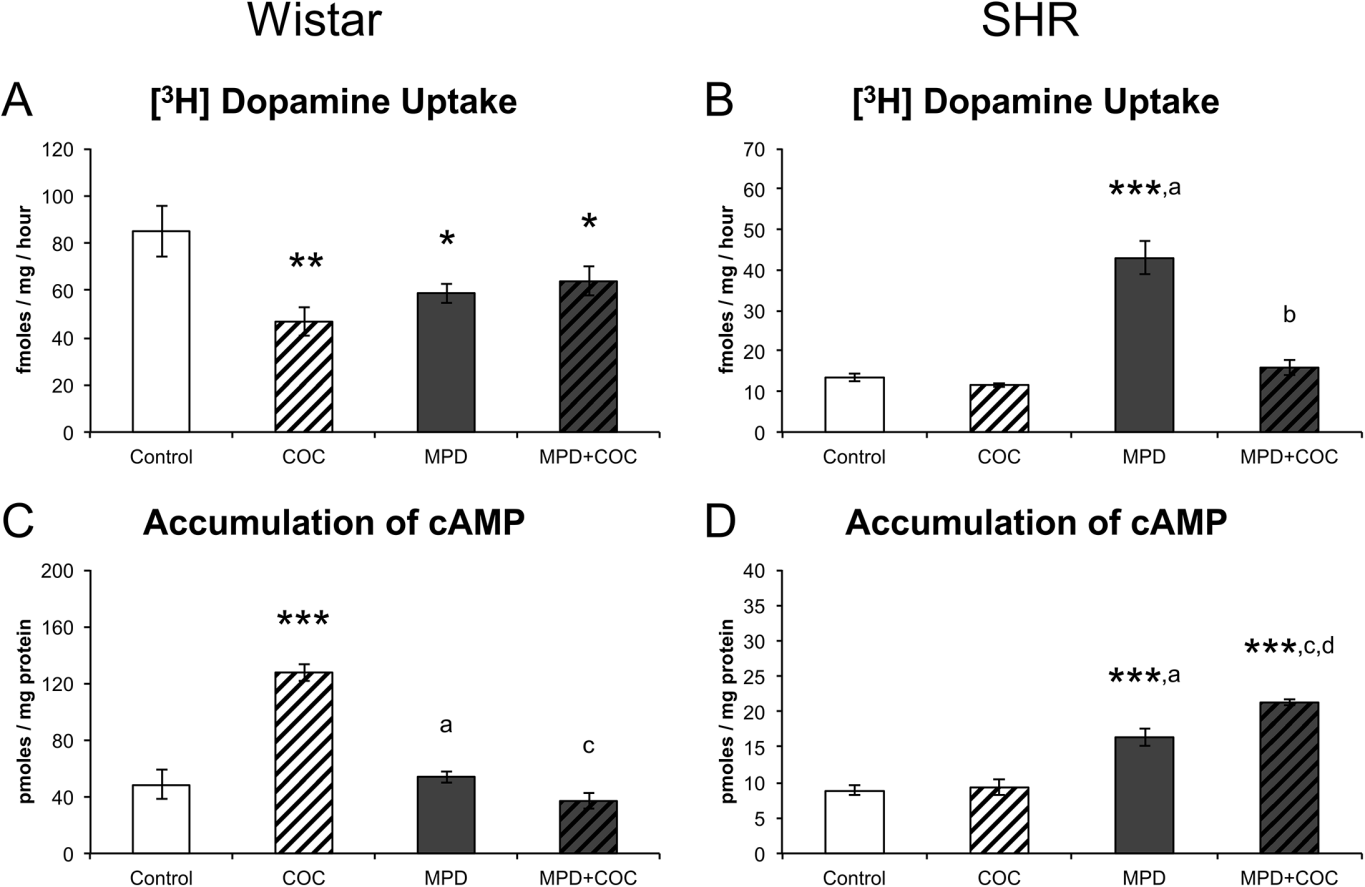
[^3^H]-Dopamine uptake and cAMP accumulation in MPD-withdrawn SHR and Wistar rats after a challenge with cocaine. Wistar (A) and SHR (B) [^3^H]-Dopamine uptake after a challenge with cocaine (10 mg/kg i.p.) in vehicle-treated and MPD-withdrawn rats (n = 5–8). Wistar (C) and SHR (D) cAMP levels in vehicle-treated and MPD-withdrawn rats after a single administration of cocaine (n = 3–6). Results are expressed as means ± S.E.M. * = p < 0.05; ** = p < 0.01; *** = p < 0.001, vs. Control. a = p < 0.01, b = p < 0.001, vs. COC. c = p < 0.01, d = p < 0.001, vs. MPD.

Regarding [^3^H]-Dopamine uptake in Wistar rats, a significant effect of group was observed (F = 4.1, d.f. = 3, p = 0.012) (Fig 6A). This effect can be explained by the fact that uptake was significantly increased in the Control group when compared to the COC (FPLSD: p = 0.002), MPD (FPLSD: p = 0.019) and MPD + COC (FPLSD: p = 0.036) groups. No differences were observed among these three groups. As for cAMP accumulation in the same strain, a significant Group effect was observed (F = 10.5, d.f. = 3, p < 0.001) (Fig 6C): cAMP levels were significantly increased in the COC group when compared to the control (FPLSD: p < 0.001), MPD (FPLSD: p = 0.002) and MPD + COC (FPLSD: p = 0.001) groups. No differences were observed among these three groups.

As in the Wistar rats, SHR ones also had marked differences among groups regarding [^3^H]-Dopamine uptake (F = 46, d.f. = 3, p < 0.001) (Fig 6B). However, in SHR rats, the group with the highest uptake values was the MPD one (FPLSD: p < 0.001 in all pairwise comparisons with Control, COC and MPD + COC groups). No differences were observed among the other groups. Finally, a significant effect of group was likewise observed regarding cAMP accumulation (F = 33, d.f. = 3, p < 0.001) (Fig 6D). In this instance, the pattern of results was somewhat more complex: MPD + COC rats had the highest values when compared to Control (FPLSD: p < 0.001), COC (FPLSD: p < 0.001) and MPD (FPLSD: p = 0.005) animals. In addition, MPD rats had higher cAMP accumulation values than Control (FPLSD: p < 0.001) and COC (FPLSD: p < 0.001) animals. No differences were observed between these last two groups.

## Discussion

Here, we have shown that SHR animals present reduced dopamine uptake and basal cAMP production in comparison to Wistar rats. We have also shown that exogenous dopamine exposure or systemic administration of cocaine are not efficient in inducing neurochemical prefrontal responses in SHR animals. Furthermore, it was also demonstrated that a 10-day withdrawal from chronic methylphenidate treatment results in increased [^3^H]-Dopamine uptake and D1-like-dependent cAMP accumulation in SHR but not in Wistar rats. Finally, we demonstrated that a single dose of cocaine is able to modulate the dopaminergic circuitry in SHR animals previously exposed to methylphenidate in a manner that differs from what was observed regarding Wistar rats.

### Differences in the Dopaminergic System of SHR and Wistar Rats

Studies addressing the hypofunction of dopaminergic system in ADHD patients and animal models are abundant in the literature [3, 5, 8, 9, 15]. Our results have shown that SHR animals present a reduced dopamine uptake when compared to Wistar rats, which could be an important aspect of the dopaminergic hypofunction described in SHR and of the development of ADHD phenotype. Mill et al [30] suggested that one possible explanation for the similarity between patients with ADHD and the SHR model could be an improper insertion of an uncoded exon on the DAT gene, which could also occur in the corresponding gene of patients with ADHD. The resulting defect in the DAT gene would be responsible for decreasing dopamine uptake, without impairing the possibility of an increased expression of the DAT protein [31, 32].

We have also noticed that exogenous dopamine stimulation does not modify cAMP levels in naïve SHR animals, a result that markedly differs from what was observed in Wistar rats. Similarly, while [^3^H]-Dopamine uptake and cAMP production were not reduced after a single administration of cocaine in VEH-treated SHR animals, Wistar neurochemical responses were strongly affected. Our data supports the hypothesis that the deficiency in the dopamine uptake system and the absence of response in the D1R-dependent cAMP production after dopaminergic stimulation in untreated SHR animals participate in the reduction of the functionality of the dopaminergic system that has been described in many studies. In addition to the low release of dopamine [12], a decrease in DAT and D1R activity in these animals could be responsible for several of the deficits already described in this strain. Additional results of our group support these findings: prefrontal cortices of VEH-treated SHR animals exposed to 100 μM SKF 81297 (a selective D1-like receptor full agonist) do not present an enhancement in cAMP levels (see S1 File).

Interestingly, Gronier [18] emphasized that, in an electrophysiological study, a considerable number of prefrontal cortex neurons were not responsive to D1-like antagonists. Also, it has been demonstrated that DAT-cocaine insensitive mutant mice, another animal model of ADHD, present altered D1R/cAMP/PKA/DARPP32 signaling [33]. Our findings indicate that DAT and D1-like receptors of SHR animals are unresponsive to stimulation with dopamine agonists, an observation that differs from what has been seen in Wistar rats. This possibility is consistent with the reduced dopaminergic function in prefrontal cortex that was previously described by Russell [8]. However, a significant response to the D1 antagonist SCH 23390 was observed in VEH-treated SHR animals at PN56. One possible explanation for this finding is that the D1 receptor is highly expressed during adolescence and that its expression tends to decrease as the animals age [34], a possibility that could be addressed in subsequent studies.

### Effects of Methylphenidate Withdrawal

Methylphenidate has been used as an effective treatment for ADHD for many years [16, 35]. Reports indicate cognitive and behavioral improvements in SHR animals chronically treated with this stimulant [36, 37]. However, the effects of methylphenidate treatment on DAT expression in the prefrontal cortex have been controversial. Although it has been previously reported that continued administration of methylphenidate alters DAT density in presynaptic membranes in prefrontal cortex of SHR animals [38], there are also findings indicating otherwise [13]. In addition, Feron et al [39] reported that children with ADHD, three months after initiation of treatment with methylphenidate, present a reduction of the dopamine transporter in the striatal system. Notwithstanding, withdrawal of methylphenidate medication in these children resulted in an increased dopamine transporter activity, verified by single-photon emission computed tomography.

Harvey et al [25] demonstrated that SHR animals exhibit a methylphenidate-induced decrease in dopamine clearance by DAT, while Somkuwar et al [13] reported that DAT Vmax is increased after chronic treatment with methylphenidate in a trafficking-independent manner. Our results demonstrated that a 24-hour withdrawal from chronic treatment with methylphenidate at the dose of 2.5 mg/kg p.o. was ineffective in changing the dopamine uptake or the DAT expression analyzed by Western Immunoblotting. However, we have also shown that a 10-day withdrawal from chronic methylphenidate administration modulates DAT activity by strongly enhancing dopamine uptake in SHR animals, without altering DAT expression in prefrontal cortex.

The absence of a significant methylphenidate effect after a 24-hour withdrawal on DAT activity does not seem to be the result of the use of an insufficient dose. For instance, we have shown that a single dose of methylphenidate (2.5 mg/kg p.o.) substantially reduces dopamine uptake in the prefrontal cortex of SHR animals, which is consistent with findings observed in the human brain [40]. Likewise, several studies reported that acute administration of psychostimulants increases dopamine overflow in the PFC [41, 42, 43, 44, 45]. Also, Yang et al [46] have shown that acute treatment with methylphenidate (2.5 mg/kg i.p.) increases stereotypic movements in different rat strains (including SHR), indicating consistent methylphenidate-induced motor effects related to the dopamine system [47].

It is well stablished that methylphenidate can act on the dopaminergic system and increase dopamine release [48], which, in turn, could influence the post-synaptic dopamine receptors expression and/or functionality. In our study, we observed that, after a long methylphenidate withdrawal, there is an enhancement of D1-like membrane receptors associated with increased cAMP levels in SHR animals, which are blocked by SCH 23390. Nevertheless, D1R expression observed by Western Immunoblotting was not affected by methylphenidate withdrawal in this strain.

It is relevant to notice that we have observed an alteration in dopamine uptake but not in D1-dependent cAMP levels after long methylphenidate withdrawal in Wistar rats. Somkuwar et al [13] reported a decrease of DAT Km in the orbitofrontal cortex of Wistar rats chronically treated with methylphenidate, with no alteration in the maximal uptake of the neurotransmitter. The observed difference in dopamine uptake could be explained by the that different brain areas were used in each study or by total the period of methylphenidate withdrawal to which the animals after were subjected. It must be pointed out that no previous study has demonstrated the effects of methylphenidate on D1-dependent cAMP levels in Wistar rats and that we have demonstrated that a 10-day methylphenidate withdrawal does not alter cAMP production in this strain.

### Cocaine Challenge after Long Withdrawal of Methylphenidate

The consequences of chronic treatment with methylphenidate or its withdrawal on cocaine abuse are poorly understood. Several studies indicate that methylphenidate treatment during adolescence in ADHD individuals may increase substance abuse liability [49, 50, 51]. However, conflicting results have also been described. Some studies support the theory that long-lasting treatment with stimulant medication does not affect the risk for substance abuse at adulthood. Despite the high rates of substance use disorders, some reports in the literature indicate that there is no correlation between the use of stimulants for ADHD treatment in young children and the risk for substance abuse during adult life [52, 53].

We demonstrated that cocaine was ineffective in inducing an enhancement of cAMP levels in VEH-treated SHR animals, markedly differing from what was observed in Wistar rats, which presented a diminished uptake and increased cAMP levels, as expected. Furthermore, we have shown that a cocaine challenge enhances cAMP levels in a D1-like dependent mechanism in SHR pretreated with methylphenidate, while it reduced cAMP levels in methylphenidate-treated Wistar rats. It is possible that the neurochemical effects observed in SHR animals are a consequence of an association between the reinforcing mechanisms of methylphenidate and the inborn maladaptive function of the SHR dopaminergic system. Our findings support the results of previous studies: Harvey et al [25] have shown that chronic oral administration of methylphenidate (1.5 mg/kg from PN28 to PN55) increases the risk for cocaine abuse (as measured by the self-administration test) in SHR animals, but not in Wistar or Wistar-Kyoto rats, while Baskin et al [54] reported that SHR animals, after a 22-day withdrawal, maintained high levels of cocaine self-administration. Here, we have shown that the 10-day methylphenidate withdrawal apparently induces an enhancement in dopamine uptake and in D1-like-dependent cAMP accumulation as well as it induces an increase in the response to cocaine in SHR animals, while causing contrasting effects in Wistar rats. Interestingly, dela Peña and collaborators [55] have demonstrated, in SHR animals under a progressive ratio schedule, that methylphenidate pre-exposure (with a clinically relevant dose) does not affect subsequent self-administration of methylphenidate, unlike what has been observed in Wistar rats, which presented an enhanced self-administration response. In addition, it was demonstrated that, under a fixed ratio schedule, SHR animals showed a slight reduction in methylphenidate self-administration, while Wistar rats were not affected.

Despite the effects found by Beaudin et al. [26], which indicates that MPD (2.5 mg/kg p.o.) ameliorates the motor dysfunction induced by manganese exposure during development, it is important to consider that the dose used in the study is above the one usually considered therapeutic. ADHD children are typically treated with 10–60 mg per day (0.5–2 mg/kg) [18]. It has been shown that methylphenidate produces an inverted dose-response curve, whereby moderate doses (1.0–2.0 mg/kg, p.o.) significantly improve delayed alternation performance, enhance cognitive function, and facilitate attention and working memory, while higher doses (2.0–3.0 mg/kg, p.o.) produce perseverative errors in many animals [56, 57, 58, 59]. Therefore, it is possible to hypothesize that the association of dose and period of methylphenidate treatment could be particularly relevant in generating the neurochemical changes observed in both strains in our study.

In conclusion, our study offers additional insight into the dopaminergic mechanisms mediating methylphenidate use during infancy and adolescence in animal models with or without the ADHD phenotype, and the neurochemical consequences at adulthood. Our results unveil functional aspects of chronic methylphenidate treatment in the prefrontal cortex of these adolescent animal models, and show how methylphenidate withdrawal causes changes in the dopaminergic system of this ADHD animal model. These findings indicate that SHR animals, after a prolonged withdrawal of methylphenidate, present increased dopaminergic sensitivity to psychostimulants, such as cocaine. This may be a possible mechanism that explains the high comorbidity between ADHD adolescent patients under methylphenidate treatment and substance abuse in adult life.

## Supporting Information

S1 Fig cAMP accumulation in MPD-withdrawn SHR rats after a challenge with SKF (n = 4–6). A significant group effect was observed (F = 31, d.f. = 3, p < 0.001) regarding cAMP accumulation, with the following rank order: MPD+SKF > MPD > SKF = Control. Results are expressed as means ± S.E.M. *** = p < 0.001, vs. Control. a = p < 0.01, b = p < 0.001, vs. SKF. c = p < 0.001, vs. MPD.
(PDF)

S1 File Text with the descriptions of “SKF Experiment”. (DOC)

S2 File ARRIVE Guidelines Checklist. (PDF)
