# Supplementary figures and images for: Long Withdrawal of Methylphenidate Induces a Differential Response of the Dopaminergic System and Increases Sensitivity to Cocaine in the Prefrontal Cortex of Spontaneously Hypertensive Rats
Source: PLoS One. 2015 Oct 28;10(10):e0141249. doi: 10.1371/journal.pone.0141249 (PMC4625026; doi:10.1371/journal.pone.0141249)

# Accumulation of cAMP

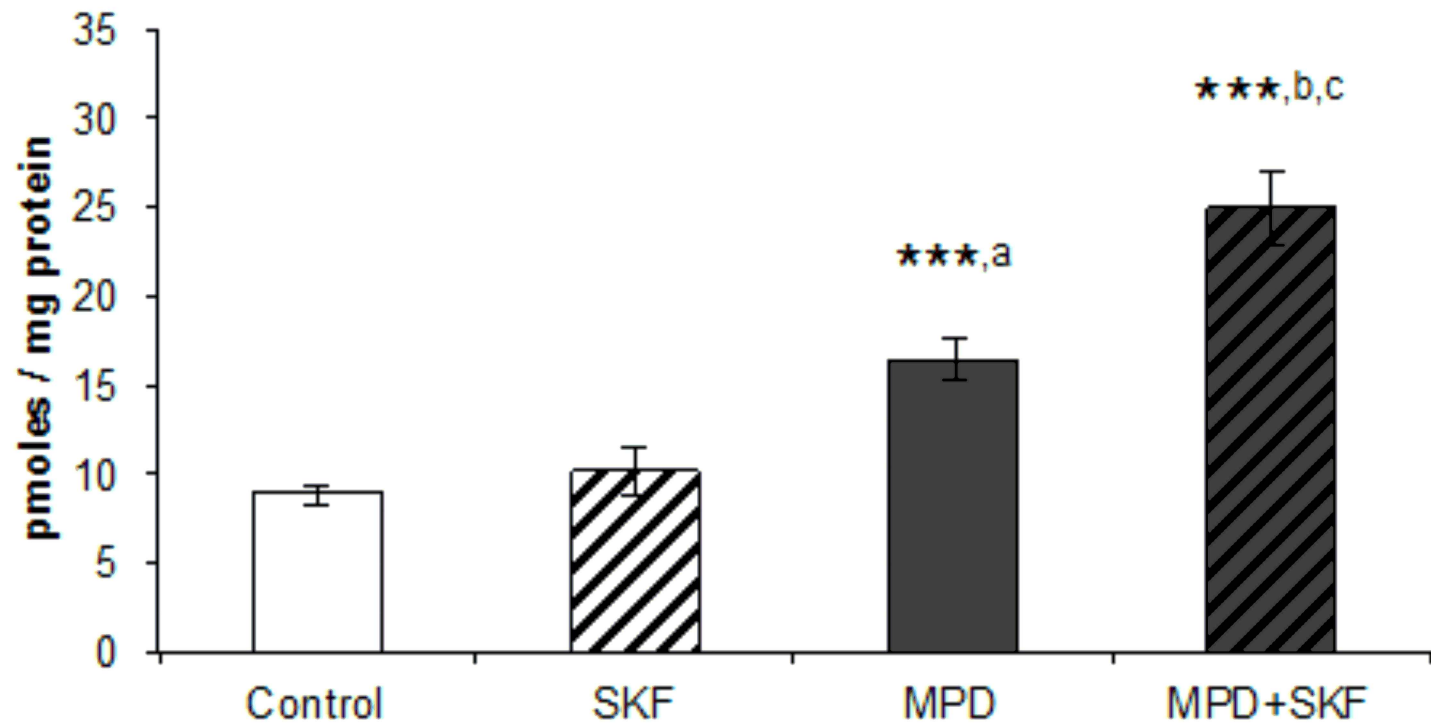

Supplement: S1 Fig — A significant group effect was observed (F = 31, d.f. = 3, p < 0.001) regarding cAMP accumulation, with the following rank order: MPD+SKF > MPD > SKF = Control. Results are expressed as means ± S.E.M. *** = p < 0.001, vs. Control. a = p < 0.01, b = p < 0.001, vs. SKF. c = p < 0.001, vs. MPD. (PDF) [file pone.0141249.s001.pdf]
