# Supplementary material for: Long Withdrawal of Methylphenidate Induces a Differential Response of the Dopaminergic System and Increases Sensitivity to Cocaine in the Prefrontal Cortex of Spontaneously Hypertensive Rats
Source: PLoS One. 2015 Oct 28;10(10):e0141249. doi: 10.1371/journal.pone.0141249 (PMC4625026; doi:10.1371/journal.pone.0141249)
Supplement: S1 File — (DOC) [file pone.0141249.s002.doc]

**Supporting Information Captions**

**S1 File:**

**SKF Experiment:** In order to evaluate whether there was an absence of dopaminergic stimuli in SHR and if methylphenidate could alter this specific pattern, we performed a cAMP assay in the presence of specific D1-like receptor full agonist SKF 81297 using vehicle-treated and 10-day methylphenidate withdrawn SHR (PN65).

*Methylphenidate treatment protocol*: Please see main text.

For cAMP accumulation, vehicle-treated and methylphenidate withdrawn prefrontal cortices of SHR were exposed to vehicle or SKF 81297 (100µM) 15 minutes before the exposure to phosphodiesterase inhibitor IMBX (0.5mM). Following IBMX exposure we proceeded with normal protocol for cAMP accumulation.

*Cyclic AMP Assay:* Please see main text.

*Statistical Analysis:* The Kolmogorov–Smirnov one sample test (K–S) was used to assess the normality of the distributions of each of the variables. A one-way ANOVA was used to analyze differences in cAMP accumulation between groups: Group (Control, SKF, MPD and MPD+SKF) was used as the between-subjects factor. The Fisher’s Protected Least Significant Difference (FPLSD) test was used post hoc. Significance is assumed at the level of p < 0.05.

cAMP accumulation in vehicle-treated SHR was not affected by SKF 81297 while it significantly enhanced cAMP levels of methylphenidate withdrawn SHR, in a similar way of cocaine. Our result corroborates our previous data indicating that naïve- or vehicle-treated SHR are unresponsive to dopaminergic stimuli and that a long withdrawal of methylphenidate is capable of reversing this response.

**S1 Fig:** cAMP accumulation in MPD-withdrawn XXX rats after a challenge with SKF (n = 4-6). A significant group effect was observed (F = 31, d.f. = 3, p < 0.001) regarding cAMP accumulation, with the following rank order: MPD+SKF > MPD > SKF = Control.Results are expressed as means ± S.E.M. *** = p < 0.001, vs. Control. a = p < 0.01, b = p < 0.001, vs. SKF. c = p < 0.001, vs. MPD.
